# Supplementary material for: Effects of Allium hookeri on gut microbiome related to growth performance in young broiler chickens
Source: PLoS One. 2020 Jan 10;15(1):e0226833. doi: 10.1371/journal.pone.0226833 (PMC6953852; doi:10.1371/journal.pone.0226833)
Supplement: S1 Table — (PDF) [file pone.0226833.s001.pdf]

S1 Table. Results of PERMANOVA of Pair-wise test for combinations groups

Pair	P-value	Pair	P-value
Leaf 0.3, Leaf 0.5	0.115	Leaf 0.5, Control	0.058
Leaf 0.3, Control	0.086	Leaf 0.5, CS	0.027
Leaf 0.3, CS	0.056	Leaf 0.5, Root 0.3	0.029
Leaf 0.3, Root 0.3	0.028	Leaf 0.5, Root 0.5	0.029
Leaf 0.3, Root 0.5	0.229	CS, Root 0.3	0.656
Control, CS	0.544	CS, Root 0.5	0.486
Control, Root 0.3	0.685	Root 0.3, Root 0.5	0.715
Control, Root 0.5	0.837		
