# Supplementary figures and images for: Effects of Allium hookeri on gut microbiome related to growth performance in young broiler chickens
Source: PLoS One. 2020 Jan 10;15(1):e0226833. doi: 10.1371/journal.pone.0226833 (PMC6953852; doi:10.1371/journal.pone.0226833)

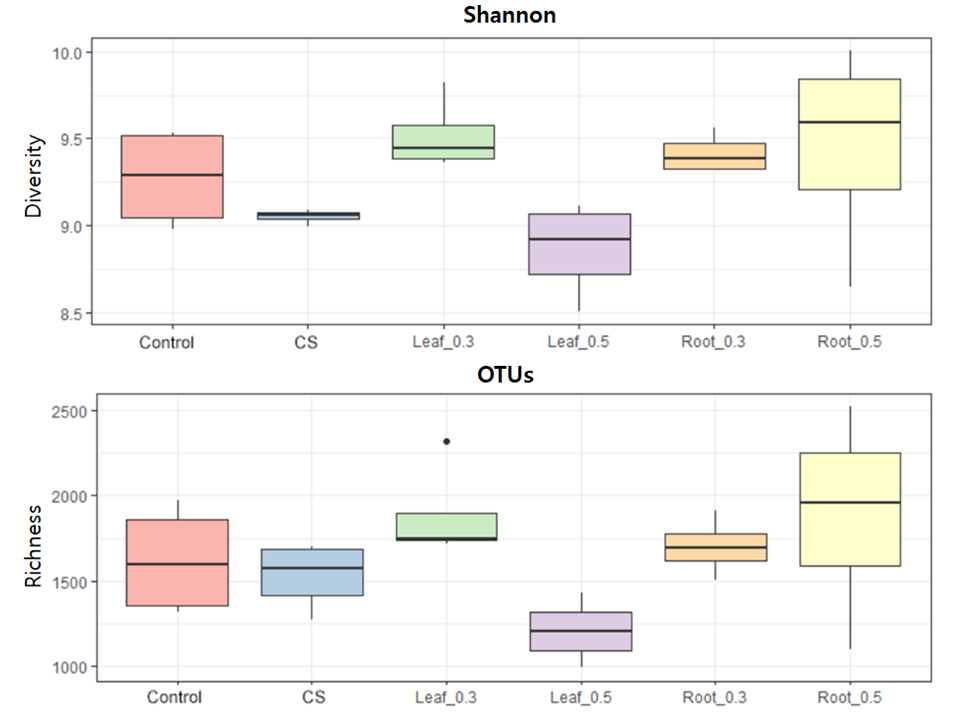

Supplement: S1 Fig — In Shannon index, a significant difference was observed between Leaf 0.3 and Root 0.3 group compared with CS (P = 0.02). In the observed OTU, Leaf 0.3 differed significantly from CS (P = 0.02). (TIF) [file pone.0226833.s002.TIF]

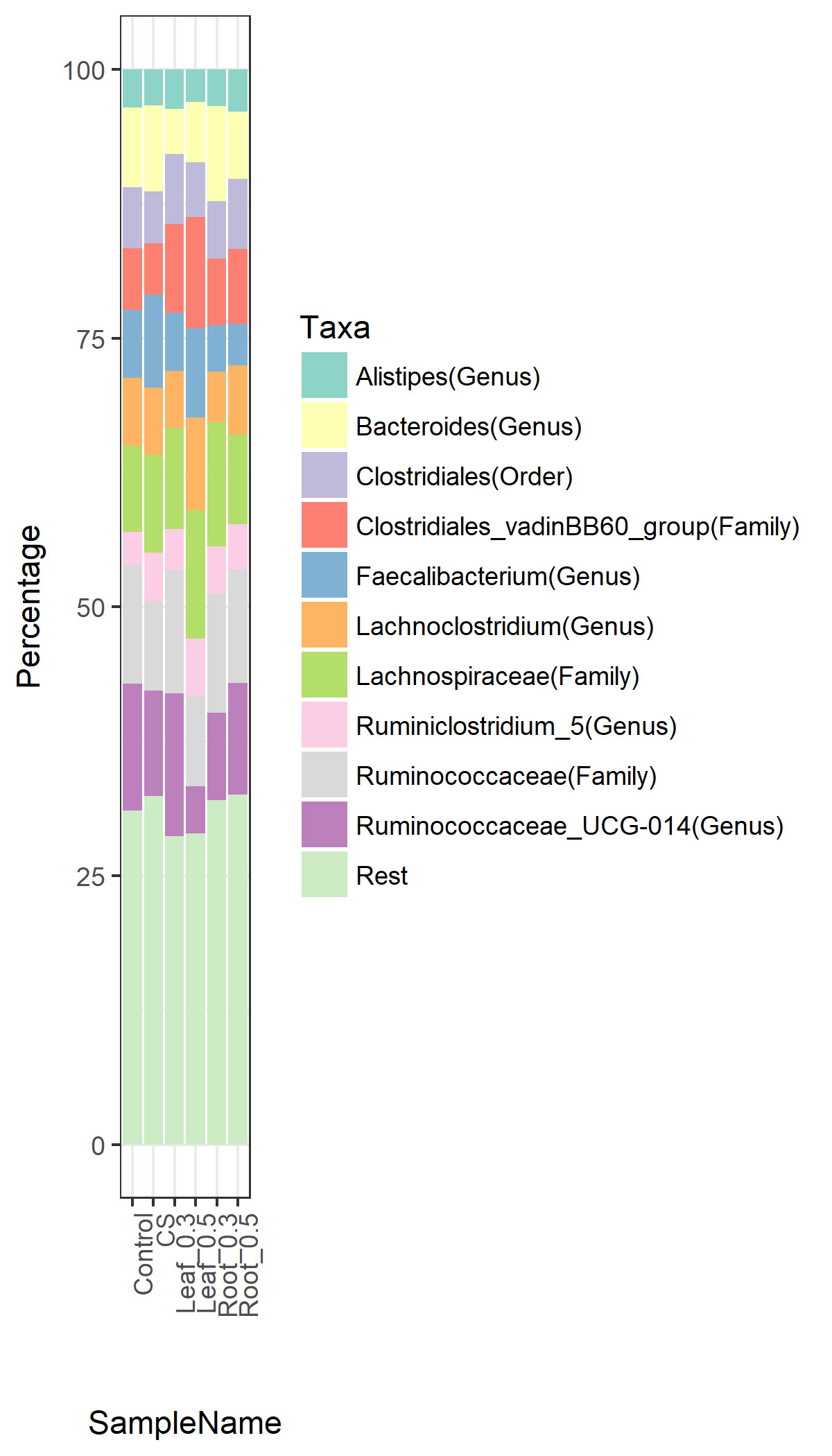

Supplement: S2 Fig — Bar plots represent the percentage (%) of average abundance among groups. (JPG) [file pone.0226833.s003.jpg]

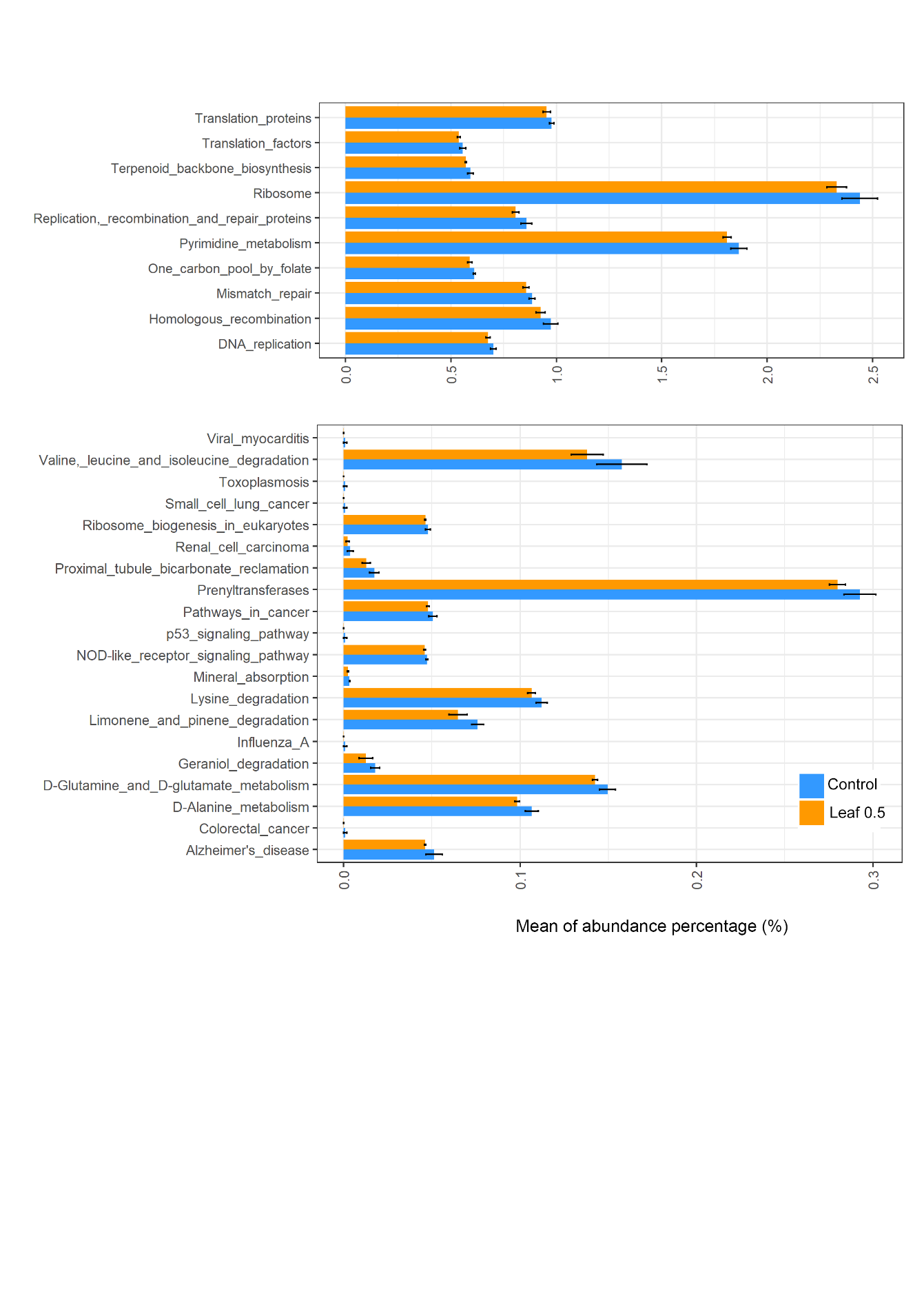

Supplement: S3 Fig — Functions were predicted by PICRUSt. All functions enriched in Control compared with Leaf 0.5 are presented (P-value < 0.05). Terms are separated into upper and lower boxes because of scale. (TIF) [file pone.0226833.s004.tif]

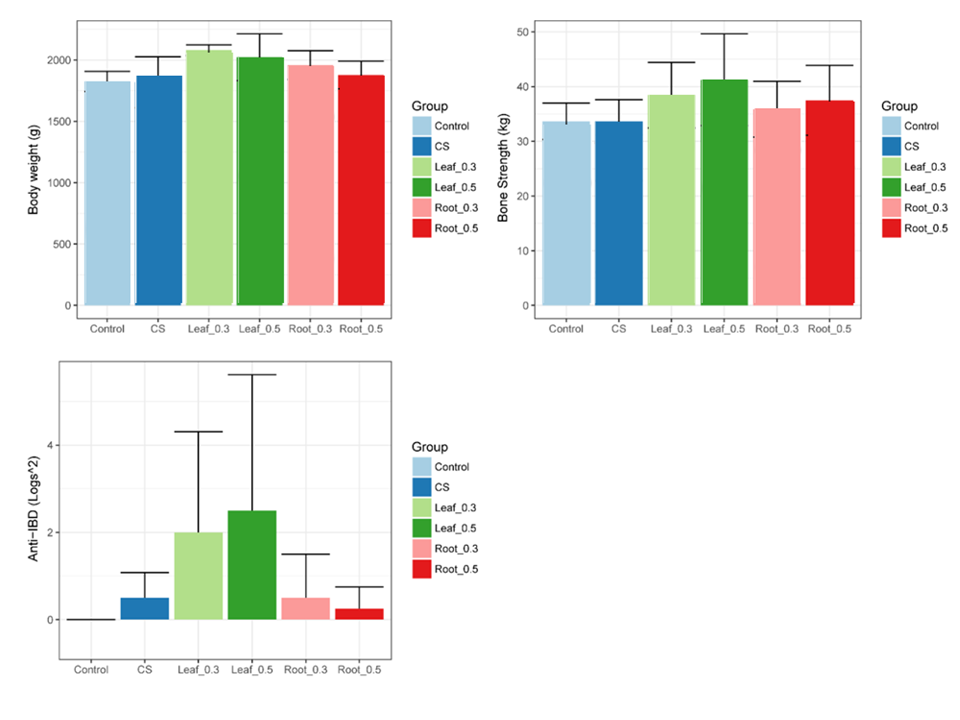

Supplement: S4 Fig — Leaf 0.3 and Leaf 0.5 showed higher body weight (P-value: 0.001 and 0.052, respectively) compared with the control group. (TIF) [file pone.0226833.s005.tif]
