# Supplementary material for: Effects of Allium hookeri on gut microbiome related to growth performance in young broiler chickens
Source: PLoS One. 2020 Jan 10;15(1):e0226833. doi: 10.1371/journal.pone.0226833 (PMC6953852; doi:10.1371/journal.pone.0226833)
Supplement: Attachment — Submitted filename: Responses to reviewers_2019 10 31-5.docx [file pone.0226833.s006.docx]

*October 31, 2019*

*Dear Dr. Juan J Loor,*

*On behalf of the authors, I would like to thank you for providing us the opportunity to improve our manuscript based on the expert reviewers’ comments and suggestions. Their constructive comments and suggestions were of great help in strengthening this manuscript.*

*1. We have provided point-by-point responses to all comments following this letter.*

*2. Most of all, this research was fully supported by the fund of Agriculture Science & Technology Development of National Institute of Agricultural Sciences (PJ01178705, PJ01327901). The government fund was used to design this study, conduct the animal studies including sample collection and analysis based on experimental results, and prepare this manuscript.*

*1) I am the corresponding author affiliated with the National Institute of Agricultural Sciences. I have submitted all the sample analyses to C&K genomics, which does not alter our adherence to PLOS ONE policies regarding sharing of data and materials including any related patents, products, or publication. I have included Ms. Bang SH and Mr. Kim BS as coauthors because they assisted with sample and data analysis, and writing of this manuscript.*

*2)For the BHNBIO, Dr. Kim and Mr. Kang from this company participated in the study design, sample collection, and writing this manuscript. They do not alter our adherence to PLOS ONE policies on sharing data and materials including any related patents, products, or publication.*

*I hope this manuscript is suitable for publication in your journal.*

*Please do not hesitate to contact me should you have any question or any other information.*

*Thank you for your kind consideration.*

*Best regards,*

Sung Hyen Lee, Ph.D.

Senior scientist, Lab leader

Functional Food & Nutrition Division, Department of Agro-food Resources

Rural Development Administration, Wanju-gun, 55365, Korea

82-063-238-3702, e-mail: [lshin@korea.kr](mailto:lshin@korea.kr) or [sunghyeonl@yahoo.co.kr](mailto:sunghyeonl@yahoo.co.kr)

Response to the Reviewer 1

**General comment of reviewer 1:**

The article has scientific merit. However, it needs some adjustments and some clarification.

**Response to general comment**

We appreciate your valid comments on our manuscript. All comments and suggests were reviewed carefully. We believe that your suggestions have greatly improved our paper. Please find the details of the revision below.

**Comment #1 of reviewer 1:**

The title should contain the word "root", not just leaf

**Responseto comment #1**

We agree with your suggestion to include the “root” in the title. We revised the title as follows: “Effects of *Allium hookeri* on gut microbiome related to growth performance of young broiler chickens”.

**Comment #2 of reviewer 1:**

Important information is missing from the summary as the main bacteria found, what were the main changes with the treatments. Still in the summary, the authors report that the main changes are involved with the 0.5% level, but I disagree, throughout the work it is possible to realize that 0.3% has a great impact on the results.In the conclusion of the summary it must be clear that the effect was of the leaf.

**Response to comment #2**

We agree with the reviewer that it is important to mention the effect of leaf 0.3%. We revised the Abstract as follows:

The composition of microbiome in the group supplemented with *A. hookeri* leaf was distinguished from that in the control group. Especially, 0.5% amounts of leaf caused higher different abundance in genera compared to 0.3% leaf. Diet of leaf 0.5% caused decreased abundance of bacteria in some genera including *[Eubacterium] nodatum group, Marvinbryantia, Oscillospira,* and*Gerlria*. The modulated gut microbiome by leaf supplement was correlated with growth traits including body weight, bone strength, and infectious bursal disease antibody. The results demonstrate that the benefit of *A. hookeri* for broiler chicken is related to the altered gut microbiome.

**Comment #3 of reviewer 1:**

At the end of the introduction the goal needs to be clearer, it went far beyond the profile of the microbiota. Several correlations were presented that were also objective.

**Response to comment #3**

We would like to thank the reviewer for the constructive comment. We revised the introduction for clarity of study goals as follows;

The objective of this study was to determine the effect of *A. hookeri* on chicken gut microbiome. We investigated the alternation of gut microbial communities in chicken by the feed of *A.hookeri* according to the tissues such as leaves and roots. We sequenced a total of 24 caecal samples derived from six groups of chickens (four samples each). The groups include chickens fed with 0.3% leaves, 0.5% leaves, 0.3% roots and 0.5% roots. Effects of *A. hookeri* as a feed additive were evaluated by comparing the *A. hookeri* diet groups with the control group (Control) or commercial supplement group (CS). We examined the altered microorganisms and their role in phenotype.

**Comment #4 of reviewer 1:**

In line 128, the authors use the term microbes, the advised term is the microorganisms please note this throughout the text.

**Response to comment #4**

We appreciate the reviewer for the constructive comment. We revised the word “microbes” to “microorganisms” in the sentences.

**Comment #5 of reviewer 1:**

Data on the composition (profile) at the genus level should be showed for all treatments this will make it easier to understand figures 2b and 2c.

**Response to comment #5**

We apologize that we did not clearly explain the abundance at the genus level in the manuscript.We included the composition at genus level and corrected the manuscript as follows.

Approximately 70% of the features have been identified at the genus level (The dominant genera in the samples are shown in S3 Fig). These features were used to determine the differential abundance of microorganisms by comparing each *A. hookeri* supplement group with the Control or CS (FDR < 0.05). For the root supplement, no differential abundance of taxa was observed.

S3 Fig. Phylum level composition.

Bar plots represents the percentage (%) of average abundance for groups.

**Comment #6 of reviewer 1:**

In the discussion, between lines 232 and 238 the authors report that A.hookeri has effect as a prebiotic, however, they do not make it clear. But if it has prebiotic action what would be the leaf component responsible for this effect? And how much of this compound is in the leaf?

**Response to comment #6**

We suggested that the prebiotic effect might be caused by polyphenols. Polyphenols, which are not absorbed in the small intestine, are hydrolyzed by the gut microbiome [1]. Polyphenol metabolites produced by gut microbiota are strongly absorbed intestinally, resulting in increased levels of healthy gut bacteria including *Bacteroides* and *Firmicutes,* and preventing the growth of pathogens. In the previous study investigating leaves, bulbs, and roots of fresh *Allium hookeri*, the leaves showed the highest levels of total phenolic compounds (240.4-276.6 mg gallic acid equivalents/100 g)[2]. We indicated the specific figures for the amount of phenolics and revised the manuscript accordingly.

Polyphenol is known to exhibit antioxidant activity that potentially prevents diseases related to oxidative stress, including cancer and cardiovascular diseases[1]. A large proportion (~90%) of absorbable polyphenols are digested by gut microbiome rather than digestive enzymes in the small intestine[3]. During the degradation of polyphenols by microbiome, intermediates including aglycones that promote various aromatic acid metabolites are produced[4]. We estimated that the altered metabolites modulate the composition of microbial communities with prebiotic effects such as antimicrobial activities against pathogens[5]. Especially, the reduction of bacteria was observed only in leaves supplement, which showed the higher levels of total phenolics (240.4-276.6 mg gallic acidequivalents/100 g) than that of roots [2]. In broiler chickens, polyphenol-rich grape increased *Enterococcus* but decreased *Clostridium*[6].

**Comment #7 of reviewer 1:**

In line 251 boiler = broiler

In line 256 the authors report the following sentence "In the present study a subset of samples from our previous study was used" two points here are crucial. First: the methodology for performance analysis should be in the material and methods as well as bone and IBD results should be presented. What statistical analyzes were used for these data? Second: If these performance data were performed in a separate experiment; Correlation between the data is not feasible because we know that the microbiota is variable.

**Response to comment #7**

We would like to thank the reviewer for the constructive comment. We agree that we used the subset of samples and discussed the variation in microbiota between individuals. However, the correlation was feasible because we used the same samples for the analysis of phenotype (body weight, bone, and IBD) and bacterial abundance.

The methodology for performance analysis as well as bone and IBD results have been presented in each appropriate section.

The method was added to the manuscript as follows.

**Performance analysis**

In the present study, body weight, bone strength, and IBD antibody level of experimental chicken were measured at 5 weeks of age [47]. Four chickens in each of the six groups similar to the average group weight were selected for the Performance analysis. Thus, a total of 24 samples (four samples from each of the six groups) were used for bone strength and IBD antibody level of blood collected from heart. Tibia strength was measured using instron (Model 3342, Inatron Universal Testing Machine, Instron Corp., Norwood, MA, USA). Effects of *A. hookeri* on antibody titer against IBD in growing chickens were evaluated by Hemmagglutination inhibition test (HI test).

**Statistical analysis for growth traits**

When we compared the growth traits between groups, t-test was performed. To analyze correlation between microbiota and growth traits, Spearman correlation was used between TMM values of each sample and growth traits (body weight, bone strength and IBD antibody). The abundance (TMM value) of significantly correlated genera was visualized in heatmap using pheatmap R package.

**Comment #8 of reviewer 1:**

The discussion about the microbiota and its effect on performance, ibd and bones was short. We know that when the immune system is stimulated the impact on performance is negative. If Parasutterella stimulates the immune system, how can it be treated for performance?

**Response to comment #8**

We agree with the reviewer for this comment. In several studies, the immune system and the body weight showed negative correlation[7]. The study did not investigate the genera which has showing correlation with both body weight and immune system.

Several studies suggested the connection between immune system and bone density mediated by the development of immune cells in the bone marrow and the role played by bone cells in health and disease[8]. The loss of bone mass triggers several inflammatory diseases [9]. *Parasutterella* showed a positive correlation with irritable bowel syndrome in patients in a previous study[10]. However, the role played by *Parasutterella* in gut pathophysiology has yet to be determined. We briefly alluded to the role of *Parasutterlla* in the Discussion.

Added References

1. Manach C, Scalbert A, Morand C, Rémésy C, Jiménez L (2004) Polyphenols: food sources and bioavailability. The American journal of clinical nutrition 79: 727-747.

2. Hwang J-S, Lee BH, An X, Jeong HR, Kim Y-E, et al. (2015) Total Phenolics, Total Flavonoids, and Antioxidant Capacity in the Leaves, Bulbs, and Roots of Allium hookeri. Korean Journal of Food Science and Technology 47: 261-266.

3. Edwards C, Havlik J, Cong W, Mullen W, Preston T, et al. (2017) Polyphenols and health: Interactions between fibre, plant polyphenols and the gut microbiota. Nutrition bulletin 42: 356-360.

4. Scalbert A, Morand C, Manach C, Rémésy C (2002) Absorption and metabolism of polyphenols in the gut and impact on health. Biomedicine & Pharmacotherapy 56: 276-282.

5. Lee HC, Jenner AM, Low CS, Lee YK (2006) Effect of tea phenolics and their aromatic fecal bacterial metabolites on intestinal microbiota. Research in microbiology 157: 876-884.

6. Viveros A, Chamorro S, Pizarro M, Arija I, Centeno C, et al. (2011) Effects of dietary polyphenol-rich grape products on intestinal microflora and gut morphology in broiler chicks. Poultry science 90: 566-578.

7. de Heredia FP, Gómez-Martínez S, Marcos A (2012) Obesity, inflammation and the immune system. Proceedings of the Nutrition Society 71: 332-338.

8. Lorenzo J, Horowitz M, Choi Y (2008) Osteoimmunology: interactions of the bone and immune system. Endocrine reviews 29: 403-440.

9. Arron JR, Choi Y (2000) Bone versus immune system. Nature 408: 535.

10. Chen YJ, Wu H, Wu SD, Lu N, Wang YT, et al. (2018) Parasutterella, in association with irritable bowel syndrome and intestinal chronic inflammation. Journal of gastroenterology and hepatology 33: 1844-1852.
